# Anaesthetic management of post-burn contractures, a recurrent challenge from oil pipeline vandalization in Nigeria: a case report

**DOI:** 10.1186/1757-1626-2-9141

**Published:** 2009-12-03

**Authors:** Abiodun Oyinpreye Jasper

**Affiliations:** 1 Department of Anaesthesia, College of Health Sciences, Delta State University, Abraka Delta State, Nigeria

## Abstract

A 13 year- old girl presented to the department with sternomental contractures as a result of facial burns from kerosene explosion. Difficult airway was envisaged. Over the period of time she developed sub-mental contracture with keloids; and was scheduled for release of contractures and flap closure.

Anaesthesia was induced with halothane and 100% oxygen. A size 3 laryngeal mask airway (LMA) was inserted and anaesthesia maintained with oxygen/nitrous oxide/halothane/muscle relaxant technique. The successful placement of LMA at 2^nd ^attempt was aided by a surgical incision on the submental contracture. Blood loss was 600 mls and a unit of packed red blood cells was transfused. She made full recovery and was discharged home after 1 month.

## Introduction

This patient had second-degree burns as a result of adulterated kerosene explosion. The scar from the burns resulted in a midline mentosternal contracture which presented some difficulty with laryngoscopy and tracheal intubation. The principles of airway management in this patient are highlighted.

## Case presentation

A 13-year-old Nigerian girl of Bini descent, who weighed 30 kg, was admitted through the Accident and Emergency Unit of the hospital. She suffered burns 2 1/2 months earlier involving the face and anterior chest wall, while filling a lit lantern with adulterated kerosene at home. Kerosene was procured from unregistered outlets commonly known as black market. She was treated earlier at a private hospital. While details of previous management were not readily made known to us, we gathered however, that she had repeated dressing of the wound before it finally healed with contractures.

She did not have physiotherapy and the sub-mental contracture resulted. Her feeding and breathing were not affected. On examination, she had a blood pressure of 90/60 mmHg [12/8 Kpa] and pulse rate of 100/minutes. The respiratory rate was 20 breaths/minutes. Air entry was adequate bilaterally and heart sounds were normal without audible murmurs on auscultation. Her haemogram revealed a packed cell volume (pcv) of 27% on the ward, after which she was transfused with two units of packed cells and her preoperative pcv increased to 39%. Serum potassium was 3.5 mmolL^-1^. The serum protein was normal Consent for surgery was obtained from the parents.

Other laboratory Investigation results were normal.

## Anaesthetic management

The history and physical examination were essentially as stated above. She has a fixed flexion deformity of the neck and cicatrized angles of the mouth, which made mouth opening inadequate. The inter-incisor distance was 2 cm while the Mallampati assessment of the airway was class IV. It was difficult to assess sternomental and thyromental distances because of the contracture. The fixed flexion deformity made head extension impossible. The ASA physical status was graded as 2.

Equipment check was done in the theatre. Difficult intubation tray was set up consisting of endotracheal tubes (size 5 and 6) laryngeal mask airway (sizes 3 and 4) gum elastic, curved and straight blades of laryngoscope, malleable stillet and facemask. Resuscitative drugs were made available i.e. adrenaline, atropine, sodium bicarbonate and hydrocortisone. The ear, nose and throat (ENT) surgeon was also in attendance if tracheostomy became necessary.

The patient was pre-medicated with atropine 0.4 mg IV after obtaining a venous access with an 18 gauge cannula. Anaesthesia was induced with halothane and 100% oxygen. The halothane was titrated between 0.5 and 3.5% using Mapleson A breathing system and a size 3 transparent face mask until loss of consciousness. Since the head could not be extended and the jaw lifted to secure the airway, a nasopharyngeal airway was inserted. Direct laryngoscopic view was attempted after the patient was properly relaxed. Laryngoscopic view and Cormack and Lehane assessment of airway was impossible; it also resulted in a failed passage of the laryngeal mask.

The surgeon was called upon to make an incision on the mentosternal scar tissue taking time to maintain homeostasis with release of contracture. This took ten minutes. Although head extension became easier at this stage, laryngoscopic view became Cormack and Lehane grade 3. A size 3 LMA was eventually passed and the cuff inflated with 20 mls of air. The lungs were ventilated easily via the LMA (secured in place by means of adhesive tape) and connected to a closed circuit with CO_2 _absorber. A bite block was inserted and intermittent pharyngeal suctioning was done. Anaesthesia was maintained with an oxygen/nitrous oxide/halothane/muscle relaxant technique with a fresh gas flow of 6 L/min [Fi0_2 _of 0.5] and 0.5% halothane. Muscle relaxation was achieved with a total of 45 mg of atracurium besylate given in divided doses. The blood pressure remained stable [110/60-100/50 mmHg], and oxygen saturation was 98-100% throughout the procedure. Intravenous pentazocine (30 mg) was given for analgesia, in two divided doses of 15 mg.

The sub-mental contractures were released with meticulous attention given to haemostasis. A split thickness skin graft was carried out on the neck and the wounds were dressed with "Sufratulle" dressing. A neck collar was placed to restrict movement, care being taken to maintain a position that would discourage further development of cicatrix. The blood loss was 600 mls as assessed by visual method. She had 500 mls of normal saline, a liter of 4.3% dextrose saline and a unit of packed red blood cells during the procedure.

Surgery lasted about two hours forty-five minutes. Anaesthetic gases were turned off after reversal of neuromuscular blockade with atropine 0.06 mg i.v. and neostigmine 1.2 mg i.v. After pharyngeal suctioning, the laryngeal mask cuff was deflated and airway removed when the patient became fully conscious in the post anaesthetic care unit. Postoperative analgesia was ensured with 15 mg of pentazocine intramuscularly 4-6 hourly. Monitoring of the patient continued into the post anaesthetic care unit with close monitoring of heart rate, blood pressure, respiratory rate and oxygen saturation. Patient was given Ceftriaxone (Rocephine), 500 mg daily intramuscularly for 5 days. On review of the wound a week later, some of the graft had taken. Dressing was continued while the possibility of a future graft was held in view. Regular dressing and physiotherapy were carried out in the post operative period. The patient was discharged a month later after admission.

## Discussion

Of recent, in Nigeria with the spate of oil pipeline vandalization and adulteration of petroleum products, there has been an increased incidence of burns from petroleum products. The incidence of burns in our environment was reported as 3.5% [1] but this has been overtaken by the recent events of explosions and adulterated petroleum products. Burns are best treated at a burns centre, where specialized staff, equipment and facilities ensure optimal treatment. The sub-mental contracture in this patient would have been prevented with proper referral.

There are two phases of burns; early and late. Each of these has distinct pathological features. The patient presented with late complications of burns i.e. contractures. The main challenge to the anaesthetist in this patent was that of airway control. It was also important that the attending surgeon understood the complementary role he had to play in maintaining the airway. This was already decided in a preoperative assessment and dialogue with the surgical team. It was also obvious that, there was a limited role for surgical tracheostomy due to the presence of the midline mentosternal contracture. The ear, nose and throat surgeons were however in attendance. In contrast, the role of the anaesthetist is that of resuscitation, respiratory care, and provision of analgesia in burns.

The contractures caused a potentially difficult airway in the patient under review by a distortion of the airway externally. Although, longitudinal alignment of the oral cavity, larynx and tracheal axis was still possible, this distortion limited access to the glottis. Difficult airway increases the risk of anesthesia with the possibility of hypoxia and increased morbidity and mortality. The incidence of difficult intubation has been reported to be 1 in 2,303 in non-obstetric surgical patients [2] and a major risk factor in an anaesthesia related death [3]. It is therefore a challenging aspect of anaesthetic practice.

The principles of management of the potentially difficult airways as in this patient involve five sequential aspects of care. These are airway assessment, mobilization of human and material resources, process of airway control by intubation or other methods, monitoring and aftercare of the patients.

It is imperative that an airway assessment is carried out preoperatively to evaluate the degree of anticipated difficulty and to aid in planning strategies for alternatives. This involves history taking, to determine the cause of the difficult airway. The history should seek to exclude previous problems of intubation during anaesthesia. The most sensitive predictor of difficult intubation is however, a past history of airway problems during anaesthesia [4].

For this patient, the presence of mento-sternal contractures indicated that endotracheal intubation would be difficult. Assessment of the patient's airway was done using Mallampati score, and graded as 4. Thyromental and sternomental distances were however difficult to assess; while the fixed-flexion neck deformity also limited jaw and neck movement. Using the Cormack and Lehane criteria at laryngoscopy, she was initially scored as 4; but this improved to 3 at the incision of the mentosternal scar by the attending plastic surgeon.

Material and human resources were mobilized to provide wide range of useful equipments, skilled assistance and Surgeons had to incise the contracture as a last resort, having been fully scrubbed and gowned. Useful equipment/alternatives to performance of difficult intubation include Augustine guide for blind intubation, the multilumen Catheter guide, and the Combitube and fibre optic laryngoscopy. Of all the Combitube is inappropriate in this patient since it is only available in a single adult size. Nasal intubation using the fibreoptic bronchoscope is of great value in many cases of difficult intubation but relies greatly on the skill and experience of the operator and can be time consuming. The equipment is not only complex but also expensive and is not available in many developing countries [5].

The routes available for airway control are subdivided into routes above the cords, (nasal and oral), and routes below the cords; cricothyrotomy and tracheostomy. The route above the cords was the more frequently used route as preferred in the patient. The laryngeal mask airway is safe for fasting patients, and its role in the difficult airway is firmly established [6]. Since invented in 1981 by British anaesthetist, Archie Brain, it has become commercially available for regular use in many hospitals and was introduced in Northern America, Japan, and Australia in 1991 [7]. LMA offers advantages over a face mask with or without an oropharyngeal airway [8] because it gives room for surgical manipulation in head and neck surgeries.

Tracheal intubation rarely causes pressure injuries to the nerves of the face, although damage to teeth and soft tissue may occur. Postoperative sore throat, a common complication of endotracheal intubation is rare with the use of LMA [9]. Increase in heart rate and blood pressure may cause cardiac ischaemia in patients with cardiac disease, while incorrect placement of the tracheal tube in the oesophagus leads to life threatening hypoxia. These risk factors are absent with the use of LMA. The possibility of oesophageal or bronchial intubation is eliminated. Insertion of LMA is achieved without much difficulty in patients in whom pre-operative assessment suggests that laryngoscopy and tracheal intubation would be difficult. This makes it is a valuable addition to the anaesthetist's equipment.

Although the LMA may make an important contribution to airway management, its use is not applicable in open lung surgery or in patients with severe obstructive airway disease. It is however useful in patients with difficult airway; and could be life saving in them. The concerns of regurgitation and aspiration have led to the invention of a Pro-Seal laryngeal mask airway (PLMA) and the Streamlined Linear of the Pharynx Airway (SLIPA). The Pro-Seal LMA (PLMA) is a new device developed with a modified cuff intended to improve the seal, and a drainage tube designed to prevent aspiration by providing a bypass channel for regurgitated gastric tube through it [10]. The passage of an orotracheal tube of adequate size e.g. 6 mm, through the LMA has been recommended as a means of achieving tracheal intubation [11] although; this was not done in this case. Capnography would also have been useful in confirming correct placement of the LMA. Complications can occur with and folding back of the mask, increasing the risk of regurgitation and aspiration [12]. The complication was absent in this patient. A method for airway access and control using routes below the vocal cords during difficult intubation include retrograde intubation and tracheostomy. Tracheostomy was however not possible in the case because of the sterno-mental contracture.

The basic principle involved in the process of airway control in the patient with a difficult airway is the preservation of spontaneous respiration. While the technique of inhalational induction can be used to facilitate intubation as was attempted in this patient, intravenous induction with thiopentone sodium may require suxamethonium chloride to facilitate tracheal intubation. The excessive potassium release associated with suxamethonium begins within three day after thermal injury [13] and may last up to ninety days. The hyperkalaemia (up to 10 mmmol/l) [13] may cause severe arrhythmia and can progress to ventricular fibrillation and cardiac arrest. Normal muscle releases enough potassium during succinylcholine-induced depolarization to raise serum potassium by 0.5 meq/L [14]. While this is usually insignificant in patients with normal baseline levels, a life threatening potassium elevation is possible in patients with burns injury. This informed the avoidance of suxamethonium for endotracheal intubation. She had serum potassium of 3.5 mmol/L. Another technique to achieve the objective of maintaining the patient's spontaneous respiration in a difficult airway is the use of ketamine as sole agent; with the use of an oropharyngeal or nasopharyngeal airway. Supplemental oxygen via a facemask is essential with ketamine anaesthesia.

The next sequence of care in difficult airway management is confirmation of correct placement of equipment used, to gain airway control. It minimizes the risk of hypoxia from misplacement. Pulse oximetry was useful in this regard in the absence of Capnography. The last sequence involved care of the airway after the period for which airway control was needed (aftercare). It evaluated the level of consciousness of the patient, presence or return of airway reflexes and the presence or absence of factors such as airway oedema and secretions, which can compromise the airway in the patient. The LMA was removed after which the patient regained full consciousness.

The monitoring of the patient and the equipment used were important to assess the adequacy of ventilation and oxygenation. Pulse oximetry is useful in the care of difficult airway by confirmation of tube placement, ventilation and oxygenation. Electrocardiography for cardiac rate and rhythm are also useful in such patients. Her blood pressure, pulse and urine output were also normal. She had 500 mls of normal saline and 1 litre of 4.3% dextrose saline and a unit of packed red blood cells intraoperatively. The requirement for early recognition of airway problems has far reaching effects on the eventual outcome of a particular airway.

## Conclusion

The anaesthetic management of this patient with burns and sub mental contractures presented a special challenge to the intubation skill of the anesthetist. It demonstrated the need for careful approach to the problem of difficult airway under anesthesia. This case demonstrated the useful role played by Laryngeal Mask in the management of difficult airway with particular reference to patients with face and neck burns.

## Abbreviations

ASA: American Society of Anesthesiology; CO_2_: Carbon Dioxide; ENT: Ear, Nose and Throat; FiO_2_: Fractional Inspired Oxygen; IV: intravenous; LMA: Laryngeal Mask Airway; PCV: Packed Cell Volume; PLMA: Pro-Seal Laryngeal Mask Airway; SLIPA: Streamlined Linear of the Pharynx Airway.

## Consent

Written informed consent was obtained from the parents of the patient for the publication of this case report. A copy of the written consent is available for review by the Editor-in-Chief of this journal.

## Competing interests

The author declares that they have no competing interests.
